# Hypokalemia : what correlation with psychotic relapse ?

**DOI:** 10.1192/j.eurpsy.2022.1169

**Published:** 2022-09-01

**Authors:** B. Abassi, E. Khelifa, I. Bouguerra, K. Nourchene, L. Mnif

**Affiliations:** 1 Razi, Skolly, Manouba, Tunisia; 2 Razi Hospital, F Adult Psychiatry Department, Manouba, Tunisia; 3 Errazi hospital-Mannouba, F, Mannouba, Tunisia; 4 Razi hospital, Skolly, Tunis, Tunisia

**Keywords:** agitation, psychotic relapse, schizophrénia, hypokalemia

## Abstract

**Introduction:**

Hypokalemia is often detected on standard biological assessments of patients hospitalized for psychiatric disorders. Many explanations are advanced by clinicians like insufficient food intake or drug effects. But what if there was a relationship between this ionic disorder and psychotic relapses?

**Objectives:**

To assess the frequency of hypokalemia in patients hospitalized for a psychotic relapse and to study its relationship with certain clinical characteristics.

**Methods:**

This is a cross-sectional study conducted over a 3-month period (july-september 2021), including 37 male patients diagnosed with schizophrenia and hospitalized in a psychiatric unit for a psychotic relapse. Patients had blood collection before medication that was sent for a complete blood count and blood chemistry testing.

**Results:**

Blood potassium level ranged from 2.92 to 4.87 mmol/L with an average of 3.74 mmol/l. Half patients ( 54.1% , N=20 ) had hypokalemia. Among them, two had electric signs on their ECG and two had physical symptoms. In patients with hypokalemia, the cause of hospitalization was the agitation in 80% of cases versus 58.8% in patients with normal potassium levels. The correlation was not significant between hypokalemia and the use of a restraint (p=0.160) or the somatic history (p=0.495).

**Conclusions:**

hypokalemia is an ionic disorder that should be detected in patients with schizophrenia. It exposes the patient to the risk of a sudden death, especially with use of antipsychotics that are at a high risk for torsade de pointes.

**Disclosure:**

No significant relationships.

